# Supplementary figures and images for: Multiscale Multiobjective Systems Analysis (MiMoSA): an advanced metabolic modeling framework for complex systems
Source: Sci Rep. 2019 Nov 18;9:16948. doi: 10.1038/s41598-019-53188-0 (PMC6861322; doi:10.1038/s41598-019-53188-0)

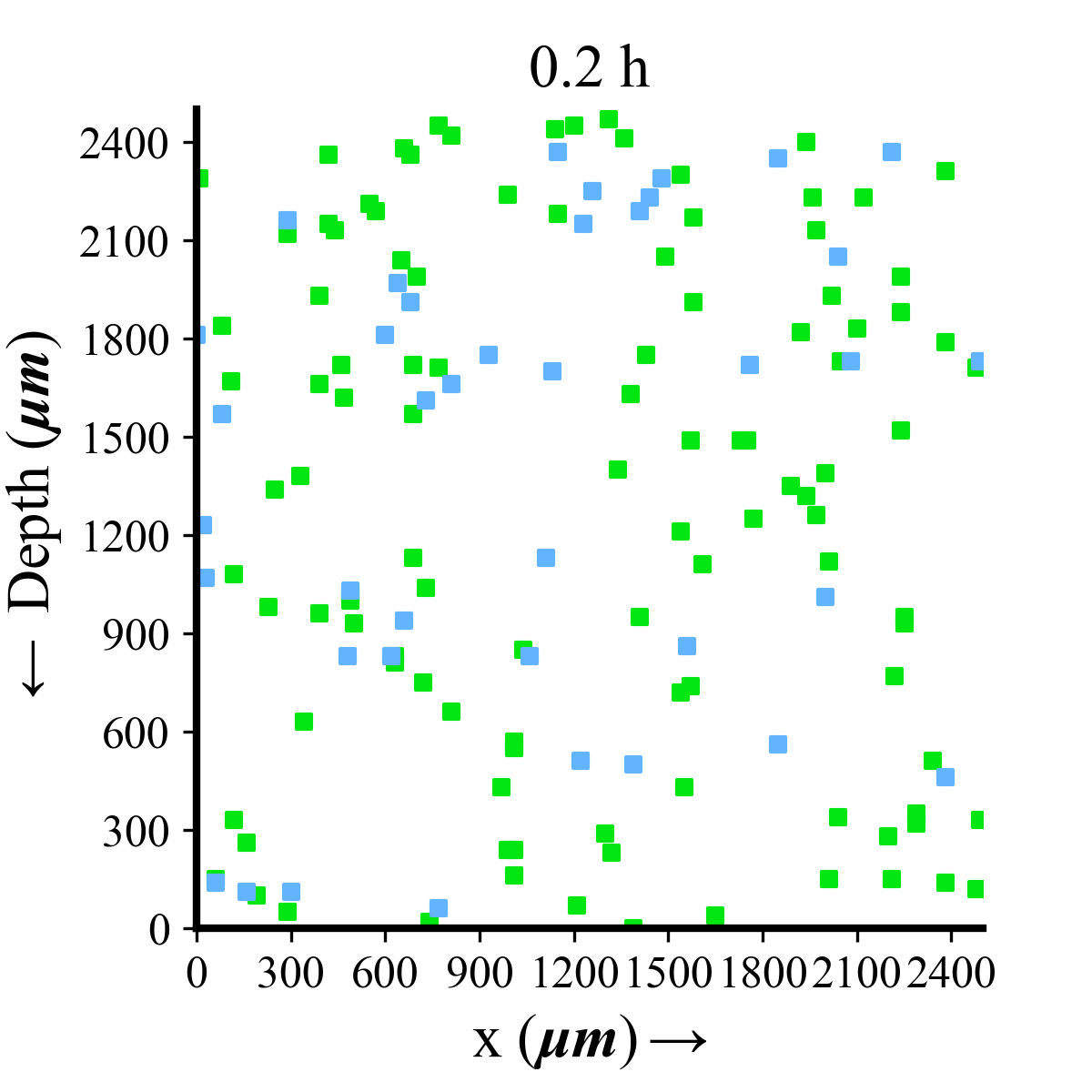

Supplement: Supplementary file 7 — Supplemental File 6 [file 41598_2019_53188_MOESM7_ESM.gif]

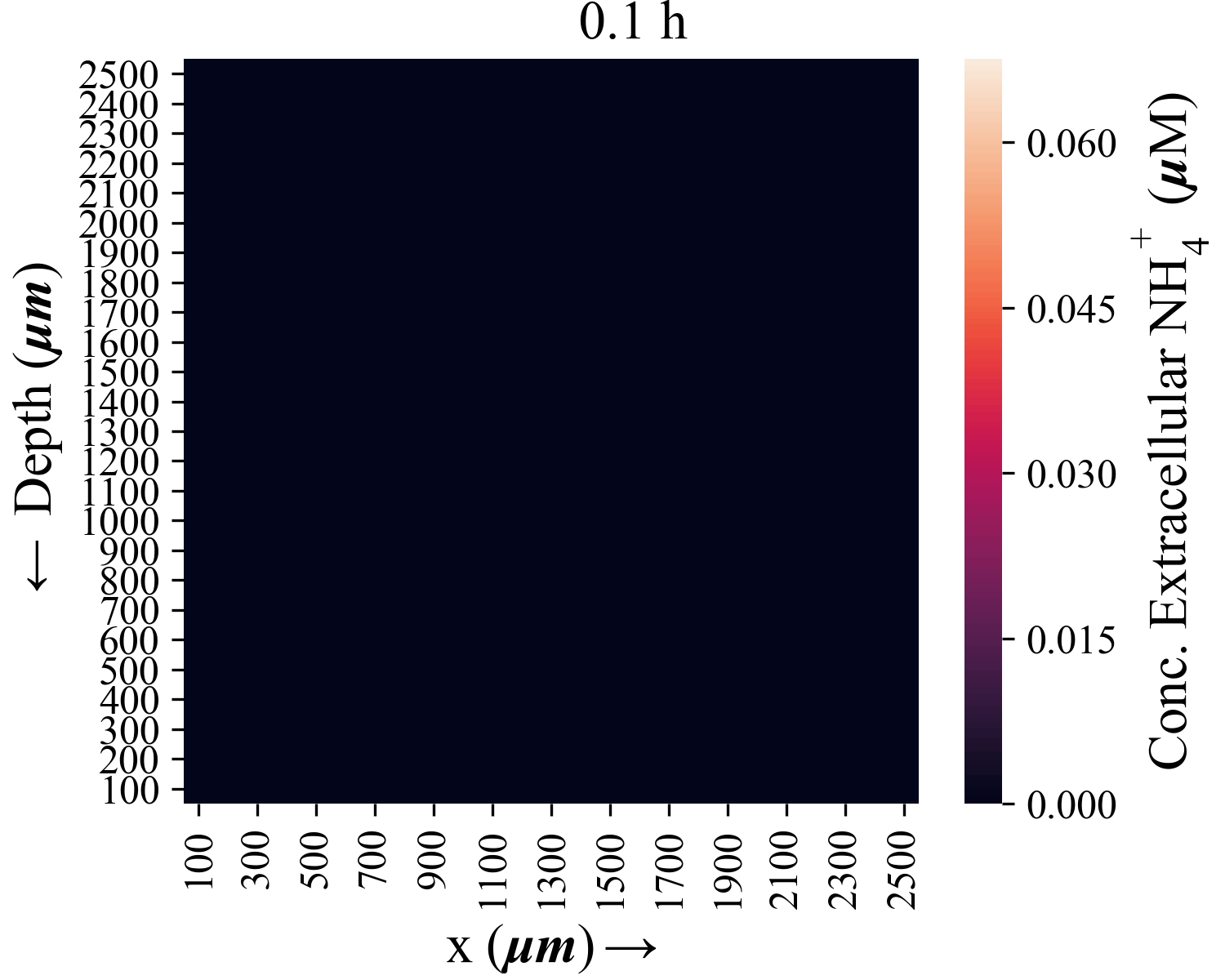

Supplement: Supplementary file 8 — Supplemental File 7 [file 41598_2019_53188_MOESM8_ESM.gif]

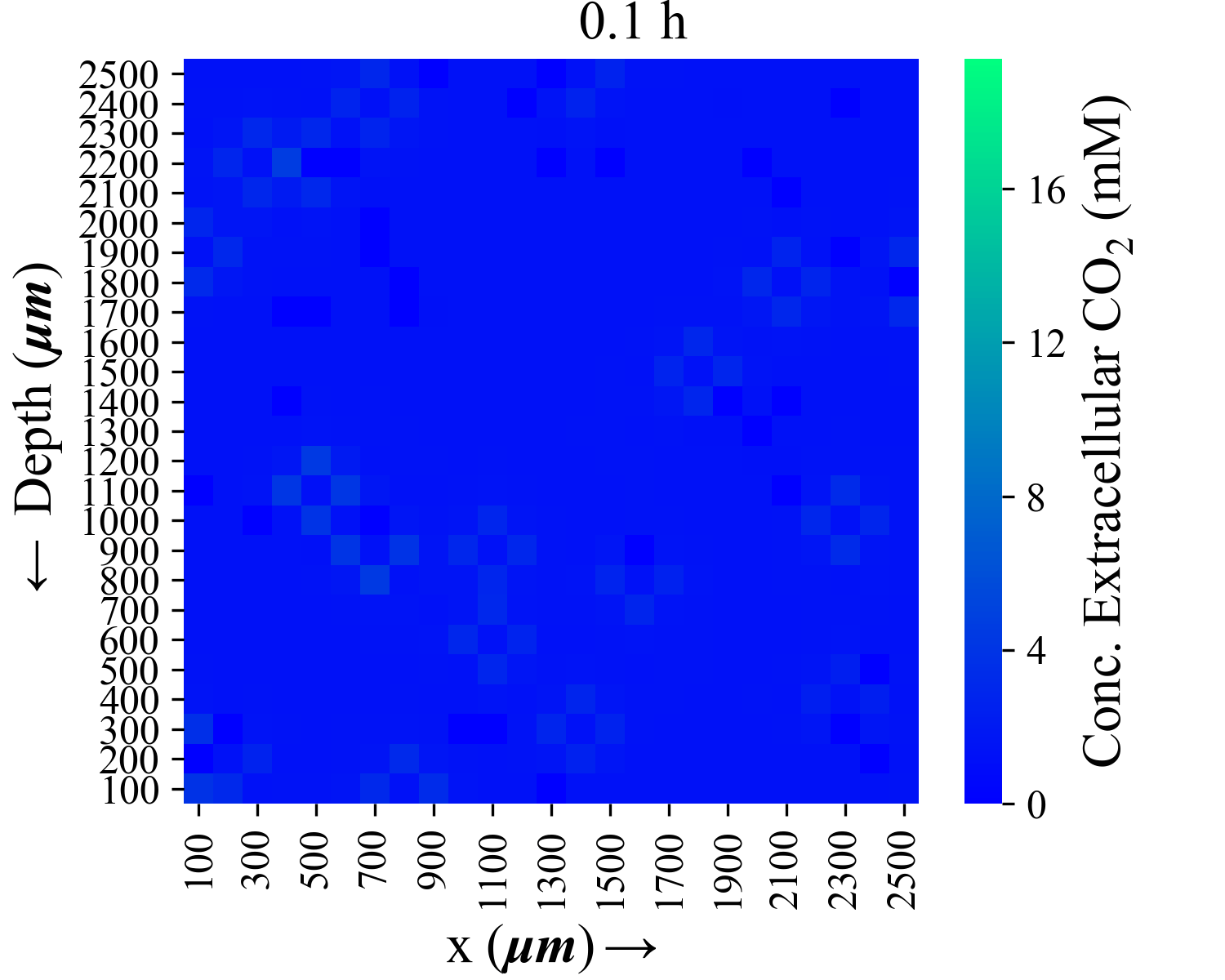

Supplement: Supplementary file 9 — Supplemental File 8 [file 41598_2019_53188_MOESM9_ESM.gif]

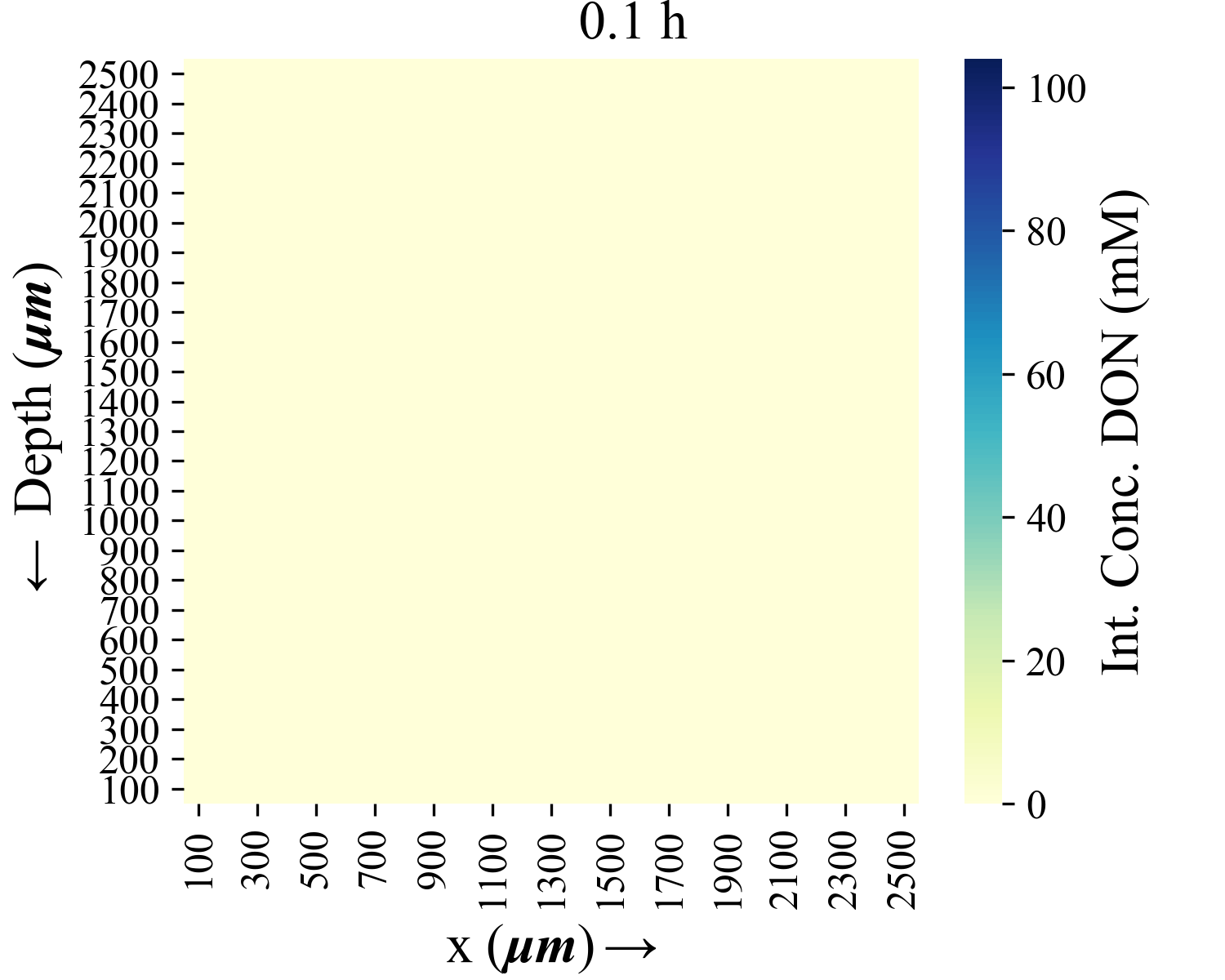

Supplement: Supplementary file 10 — Supplemental File 9 [file 41598_2019_53188_MOESM10_ESM.gif]

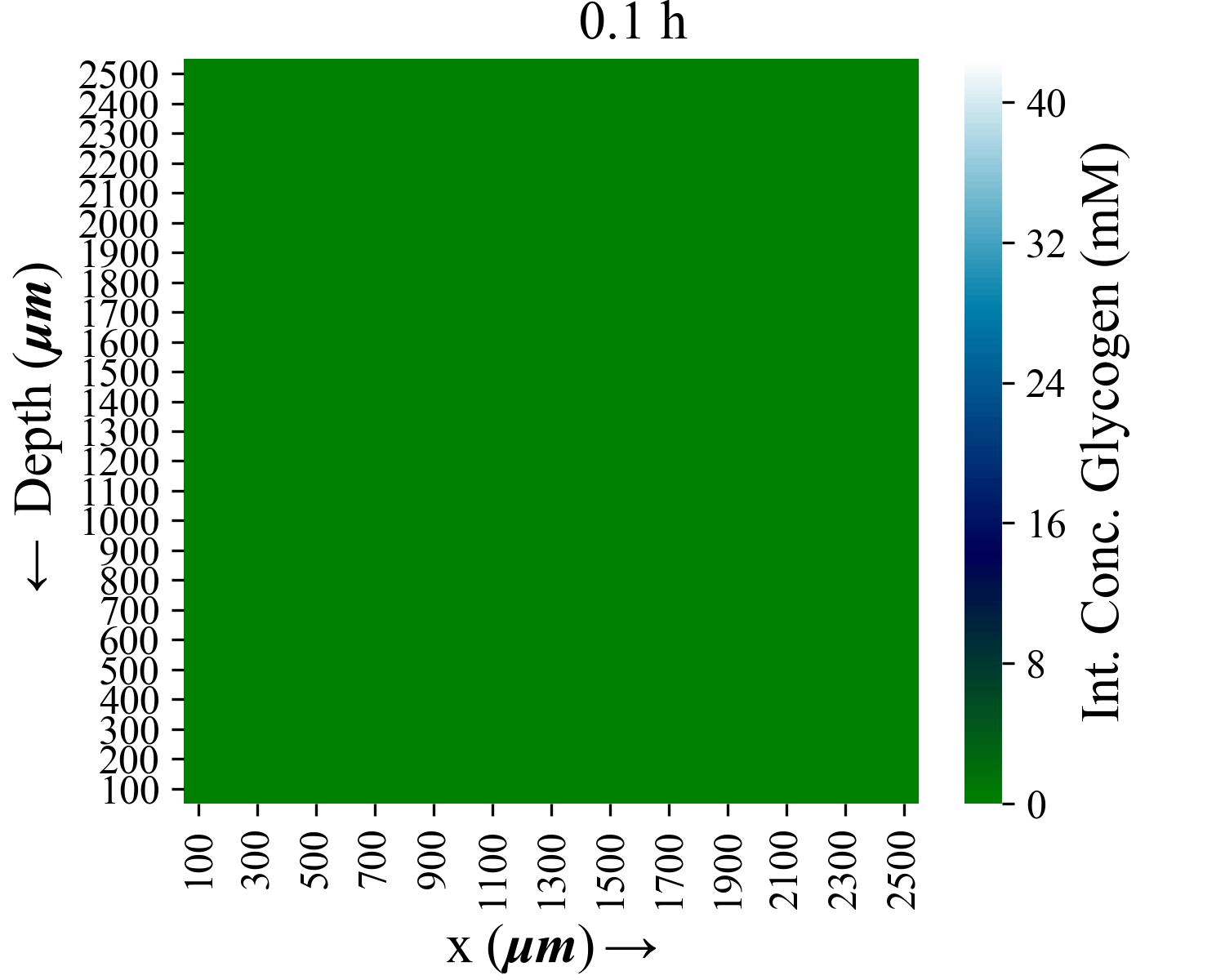

Supplement: Supplementary file 11 — Supplemental File 10 [file 41598_2019_53188_MOESM11_ESM.gif]

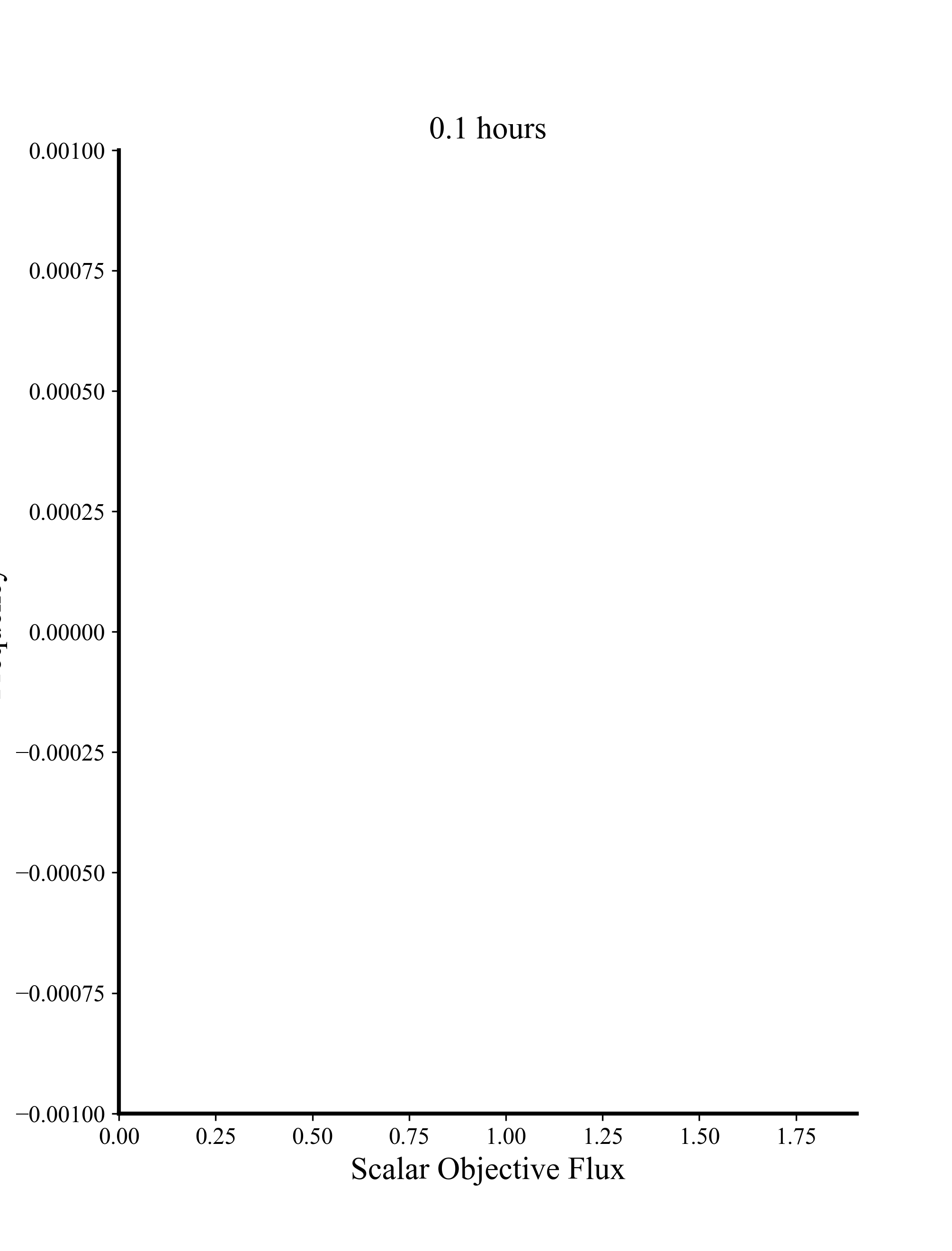

Supplement: Supplementary file 13 — Supplemental File 12 [file 41598_2019_53188_MOESM13_ESM.gif]
