# Supplementary material for: Multiscale Multiobjective Systems Analysis (MiMoSA): an advanced metabolic modeling framework for complex systems
Source: Sci Rep. 2019 Nov 18;9:16948. doi: 10.1038/s41598-019-53188-0 (PMC6861322; doi:10.1038/s41598-019-53188-0)
Supplement: Supplementary file 2 — Supplemental File 1 [file 41598_2019_53188_MOESM2_ESM.zip › iTery101/docs/index.html]

iTery101

This is the documentation for the iTery101 model...
